# TAVI in Complex Dual-Level Obstruction: A Case of Severe Aortic Stenosis and Subaortic Membrane

**DOI:** 10.14797/mdcvj.1539

**Published:** 2025-05-12

**Authors:** Salem Assiri, Mohammed S. Alshammakh, Sultan M. Alzahrani, Moath Said Alfawara, Khaled Al-Shaibi

**Affiliations:** 1 King Fahad Armed Forces Hospital, Ministry of Defense Health Services, Jeddah, Saudi Arabia

**Keywords:** transcatheter aortic valve implantation, TAVI, subaortic membrane, aortic stenosis, subaortic stenosis, interventional cardiology, valve disease management

## Abstract

This case addresses the challenges of treating patients with both severe aortic stenosis and subaortic stenosis. In this combined condition, transcatheter aortic valve implantation (TAVI) remains an off-label application, particularly in the presence of a subaortic membrane.

Our multimodal imaging approach that incorporates echocardiography, cardiac computed tomography, and fluoroscopic guidance demonstrates the successful application of TAVI in a high-risk clinical scenario. The results underscore the potential of TAVI as a viable alternative to traditional surgical aortic valve replacement for patients with dual-level obstruction who are not candidates for open surgery.

## Introduction

Transcatheter aortic valve implantation (TAVI) is a well-established and widely recognized treatment modality for patients with severe aortic stenosis (AS).^1^ However, its application in cases of combined aortic valve stenosis and subaortic stenosis (SAS), particularly involving a subaortic membrane, remains off-label. Our case offers compelling evidence supporting the safety and feasibility of TAVI in high-risk surgical candidates with complex anatomical conditions, contributing significantly to the expanding body of literature on this topic.

We utilize a multimodal imaging approach, which incorporates echocardiography, cardiac computed tomography (CT), and fluoroscopic guidance, to demonstrate the successful application of TAVI in this difficult clinical scenario. The results underscore the potential of TAVI as a viable alternative to traditional surgical aortic valve replacement for patients with dual-level obstruction who are not candidates for open surgery.

## Discussion

We report the case of an 89-year-old male who presented with chest pain, exertional dyspnea (NYHA class III), and recurrent syncopal episodes. He had a medical history significant for coronary artery disease, type II diabetes mellitus, end-stage renal disease requiring hemodialysis, and paroxysmal atrial fibrillation as well as a history of frequent hospitalizations for pulmonary edema within the preceding year.

**Figure 1:**
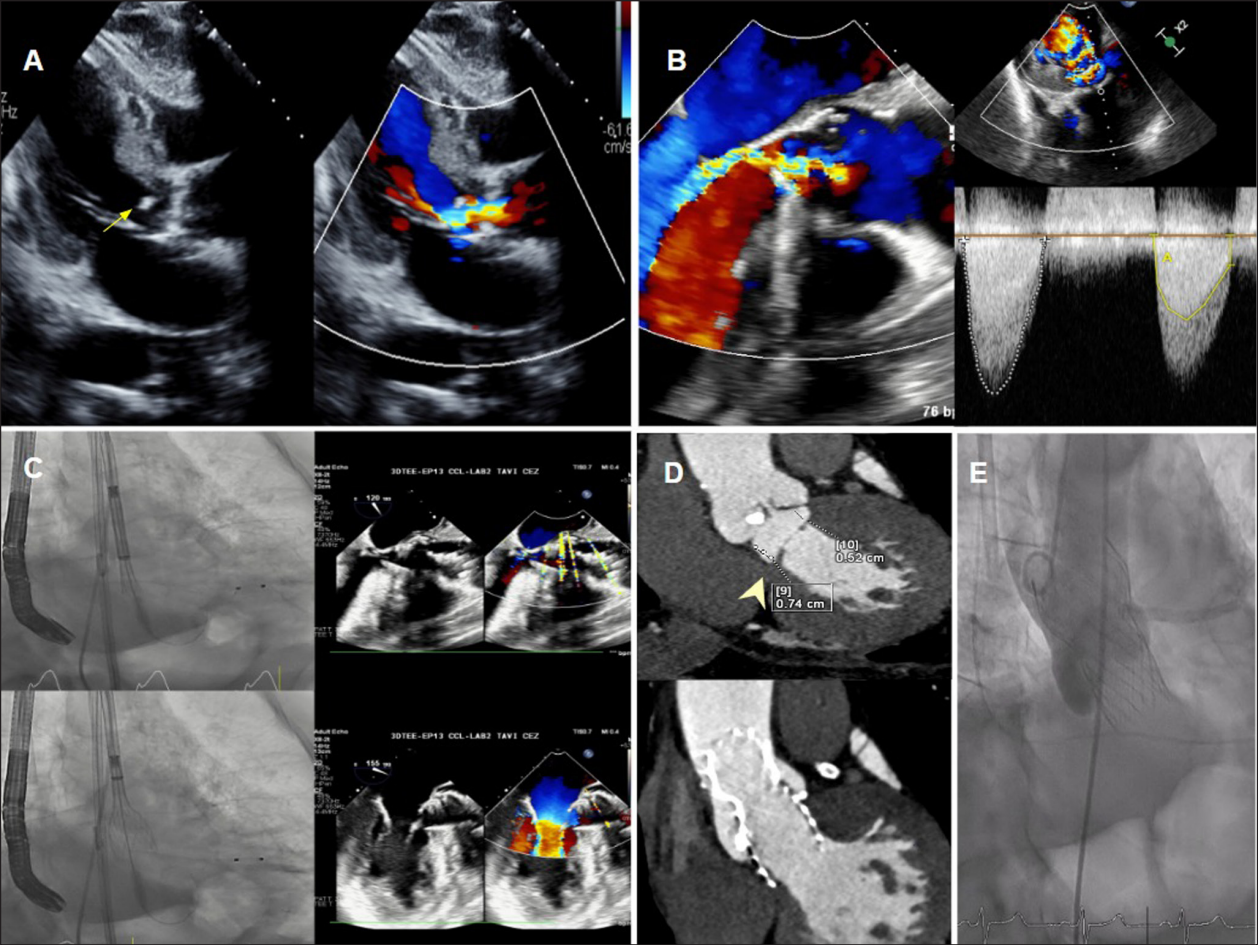
**(A)** Transthoracic echocardiography parasternal long-axis view showing subaortic membrane (arrow) with flow acceleration in the left ventricular (LV) outflow tract and below the aortic valve. **(B)** Transesophageal echocardiography (TEE) long-axis view showing subaortic membrane with flow acceleration in the LV outflow tract and below the aortic valve, continuous-wave Doppler showing a double-envelope pattern. The inner envelope on the right side of the image (yellow) represents stenosis at the subaortic membrane, while the outer envelope on the left side of the image (white) represents stenosis at the aortic valve. **(C)** A 26-mm Evolut™ PRO+ transcatheter aortic valve implantation (TAVI) was deployed with TEE guidance to position the valve precisely at the subvalvular ridge. **(D)** Cardiac computed tomography (CT) multiplanar reconstruction shows a subaortic membrane (arrowhead), and a 1-year post-TAVI CT demonstrates a well-seated valve covering most of the left ventricular outflow tract (LVOT), including the previously identified subaortic membrane. **(E)** Final aortography shows a well-seated valve in the LVOT covering the subaortic membrane, with no paravalvular leak.

Transthoracic echocardiography (TTE) demonstrated severe AS with a calcified subaortic ridge (subaortic membrane) (Figure 1A). The aortic valve area was calculated at 0.74 cm², with an indexed aortic valve area of 0.48 cm². The mean pressure gradient (MPG) across the aortic valve was 41 mm Hg, while the MPG across the subaortic membrane was 33 mm Hg. Moderate aortic regurgitation and a preserved ejection fraction of 50% were also noted. Subsequent transesophageal echocardiography (TEE) confirmed the presence of subaortic membrane with an MPG of approximately 31 mm Hg. Continuous-wave Doppler revealed a double-envelope pattern, with the inner envelope representing the gradient at the subaortic membrane and the outer envelope representing the gradient across the aortic valve (Figure 1B, Video 1). Cardiac CT TAVI protocol revealed a fibromuscular subaortic ridge, with a distance of 5 to 7 mm between the membrane and the aortic valve (Figure 1D).

**Video 1:** Transesophageal echocardiography showing subaortic membrane with flow acceleration in the left ventricular outflow tract and below the aortic valve; also see at https://youtube.com/shorts/-xcWHpuayeA.

Given the patient’s prohibitive surgical risk, the heart team opted for a percutaneous approach using a self-expandable transcatheter valve. The procedure was performed under general anesthesia using fluoroscopic and TEE guidance. Balloon valvuloplasty was performed with a 20-mm Z-Med balloon (NuMED), and a 26-mm Evolut™ PRO valve was deployed targeting placement at the subvalvular ridge. Post-dilatation was performed using a 20 x 40-mm Cristal balloon (Balt) (Figure 1C). Final supravalvular aortography confirmed proper valve positioning without paravalvular leakage or interference with the mitral valve (Figure 1E, Video 2). The MPG improved to 6 mm Hg. The procedure was well-tolerated, no pacemaker implantation was required as the baseline, and post implantation electrocardiogram revealed unchanged incomplete left bundle branch block. The patient was discharged after experiencing significant relief of symptoms.

**Video 2:** Aortography showing a well-seated valve in the left ventricular outflow tract covering subaortic membrane with no paravalvular leak; also see at https://youtube.com/shorts/kMOCuulhQ8I.

At the 6-month follow-up, TTE demonstrated stable valve positioning, effectively covering the left ventricular outflow tract and the subaortic membrane, with no encroachment on the anterior mitral valve leaflet and no significant flow acceleration in the left ventricular outflow tract. The aortic valve peak gradient was 14 mm Hg, with an MPG of 7 mm Hg, and only trace paravalvular flow posteriorly was observed (Video 3). A 1-year follow-up cardiac CT study confirmed well-functioning, sustained valve stability and proper seating (Figure 1D).

**Video 3:** Transthoracic echocardiography post transcatheter aortic valve implantation; also see at https://youtube.com/shorts/C-q8TdWPsW4.

## Conclusion

To our knowledge, only two cases have previously reported TAVI in SAS. Finkelstein et al. successfully treated a 91-year-old woman with combined AS, SAS, characterized by basal septal hypertrophy, and systolic anterior motion (SAM) of the mitral valve, using TAVI.^2^ Similarly, Alzubi described a 75-year-old male with a history of surgical resection of a subaortic membrane who presented with recurrent SAS, a type B aortic dissection, and moderate aortic regurgitation, and was successfully treated with TAVI (Miami Valve 2023).^3^

Our case adds to the growing body of evidence supporting the feasibility of TAVI in anatomically complex cases of SAS in high-risk surgical candidates. Given the rarity of such cases in the literature, we believe this report will be of substantial interest to clinicians in the fields of interventional cardiology and valve disease management. Further studies are warranted to better understand outcomes and refine management strategies in this subset of patients.
